# Causal Effects of Plasma Metabolites on Intervertebral Disc Degeneration and Low Back Pain: A Mendelian Randomization Study

**DOI:** 10.3390/metabo16090673

**Published:** 2026-09-13

**Authors:** Shouhe Zhu, Chuanzhi Tang, Weiguo Ding, Junxia Wen, Xinwei Xu, Xinhui Wang, Weixing Xu

**Affiliations:** 1 College of Integrated Traditional Chinese and Western Medicine Clinical Medicine, Tongde Hospital of Zhejiang Province, Zhejiang Chinese Medical University; Hangzhou 310012, China; 202511120811068@zcmu.edu.cn (S.Z.); 202411120811033@zcmu.edu.cn (C.T.); 2 Tongde Hospital of Zhejiang Province, Zhejiang Chinese Medical University, Hangzhou 310012, China; 3 Institute of Nutrition and Food Safety, School of Public Health, Zhejiang University School of Medicine, Hangzhou 310058, China

**Keywords:** Mendelian randomization, plasma metabolites, metabolite ratios, intervertebral disc degeneration, low back pain, causal inference

## Abstract

Background: Metabolic disturbances have been implicated in intervertebral disc degeneration (IVDD) and low back pain, but the contribution of circulating metabolites remains uncertain. Objectives: We examined associations of genetically proxied plasma metabolites and metabolite ratios with IVDD outcomes defined at different anatomical locations and with a related pain phenotype. Methods: Summary statistics for metabolite exposures were obtained from the Canadian Longitudinal Study on Ageing (CLSA) plasma metabolome genome-wide association study (GWAS). The outcomes were Intervertebral Disc Degeneration of Cervical Spine (IVDD_Cervical), Intervertebral Disc Degeneration of Thoracic, Thoracolumbar, and Lumbosacral Spine (IVDD_TTL), and Low Back Pain (Pain_Lowback). IVDD_Cervical and IVDD_TTL represented IVDD outcomes defined at different anatomical locations of the spine, whereas Pain_Lowback was analysed separately as an IVDD-related pain phenotype. One genome-wide significant sentinel single-nucleotide variant (SNV) was used for each exposure, and Mendelian randomization (MR) estimates were calculated with the Wald ratio. The within-outcome Bonferroni threshold was 6.25 × 10^−4^. Results: Eighty metabolites and metabolite ratios were evaluated. Two associations were significant, both for IVDD_TTL. Higher genetically proxied dimethylarginine (SDMA + ADMA), mapped to DDAH1, was associated with lower IVDD_TTL risk (odds ratio [OR] = 0.80; 95% confidence interval [CI]: 0.72–0.90; *p* = 2.5 × 10^−4^), whereas higher alpha-hydroxyisovalerate, mapped to LDHA, was associated with higher risk (OR = 1.08; 95% CI: 1.04–1.13; *p* = 4.6 × 10^−4^). No significant association was identified for IVDD_Cervical or Pain_Lowback; 23 associations were classified as suggestive significant. DrugBank annotation identified compounds interacting with DDAH1 and LDHA. Conclusions: The results highlight DDAH1-linked methylarginine metabolism and LDHA-linked glycolytic and lactate-related biology as candidate pathways for IVDD_TTL. Findings for IVDD_Cervical, Pain_Lowback, and cross-outcome overlap remain exploratory.

## 1. Introduction

Low back pain is a leading cause of disability worldwide and creates a substantial health burden [1]. Its clinical presentation is heterogeneous, with contributions from multiple anatomical structures and biopsychosocial factors [2]. Intervertebral disc degeneration (IVDD) is an important structural correlate of back- and neck-related pain, but cervical disc disorders, thoracic/thoracolumbar/lumbosacral disc disorders, and low back pain represent different clinical constructs. They differ in anatomical location, diagnostic coding, symptom generation, and biological interpretation. Analyses that preserve these distinctions are therefore more informative than treating low back pain as an IVDD subtype.

IVDD involves inflammatory signalling, extracellular matrix remodelling, oxidative stress, immune-cell activity, and loss of disc-cell homeostasis. Macrophage-driven inflammatory microenvironments can influence matrix metabolism, vascularization, innervation, and cellular responses during degeneration [3]. Metabolic stress is closely related to these processes because the largely avascular disc relies on nutrient diffusion and adaptation to hypoxia and limited substrate availability.

Nucleus pulposus cells have specialised metabolic requirements within this environment. Redundancy among glucose transporters helps maintain glucose-dependent metabolism under hypoxic and glycolytic conditions [4]. Mitochondrial quality-control mechanisms also influence oxidative stress, autophagy, cell survival, and matrix regulation during IVDD [5]. These observations provide a biological basis for examining whether circulating metabolic traits are associated with disc-related phenotypes.

Observational metabolomic studies cannot readily separate antecedent metabolic changes from consequences of degeneration or pain. Their findings may also be affected by age, adiposity, lifestyle, inflammation, medication use, disease severity, and reverse causation. Age and sex are additional sources of biological heterogeneity because both can shape circulating metabolic profiles and are also related to the occurrence and clinical expression of IVDD and pain phenotypes. Accordingly, differences in age and sex distribution across cohorts may modify observed metabolite–phenotype associations and should be considered when interpreting observational metabolomic evidence. Mendelian randomization (MR) uses germline variants associated with an exposure as instrumental variables and can complement observational evidence when its assumptions are satisfied [6]. The Strengthening the Reporting of Observational Studies in Epidemiology using Mendelian Randomization (STROBE-MR) recommendations provide a framework for transparent design and reporting [7,8].

Large metabolite genome-wide association studies (GWASs) now support systematic MR analyses. A 2023 study mapped genetic associations for 1091 plasma metabolites and 309 biologically constrained ratios [9], extending earlier work on more than 400 blood metabolites [10]. Despite this progress, associations of genetically proxied plasma metabolites with IVDD at different anatomical locations and with related pain remain incompletely characterised. We therefore examined 80 plasma metabolites and metabolite ratios in relation to Intervertebral Disc Degeneration of Cervical Spine (IVDD_Cervical), Intervertebral Disc Degeneration of Thoracic, Thoracolumbar, and Lumbosacral Spine (IVDD_TTL), and Low Back Pain (Pain_Lowback) using a uniform single-sentinel design. IVDD_Cervical and IVDD_TTL represented IVDD outcomes defined at different anatomical locations of the spine, whereas Pain_Lowback was analysed separately as an IVDD-related pain phenotype. The outcomes were analysed separately, and selected signals were linked to annotated effector genes and DrugBank entries to support biological interpretation and experimental follow-up.

## 2. Method

### 2.1. Data Sources and Study Design

A two-sample MR study was conducted to examine associations of genetically proxied plasma metabolites and metabolite ratios with two anatomically defined IVDD outcomes and the related pain phenotype Pain_Lowback. Exposure summary statistics were obtained from the European ancestry Canadian Longitudinal Study on Ageing (CLSA) plasma metabolome GWAS (*n* = 8299; GWAS Catalogue accessions GCST90199621–GCST90201020) [9]. Outcome summary statistics were obtained from the FinnGen R12 and UK Biobank meta-analysis [11]. IVDD_Cervical comprised cervical disc disorders with cervicalgia and cervicothoracic disc disorders (22,115 cases and 676,210 controls); IVDD_TTL comprised thoracic, thoracolumbar, and lumbosacral disc disorders (68,486 cases and 676,210 controls); and Pain_Lowback was analysed separately as an IVDD-related pain phenotype (55,116 cases and 676,210 controls). Exposure and outcome data were restricted to European ancestry populations or aligned as closely as possible with them. The use of separate CLSA exposure data and FinnGen/UK Biobank outcome data made substantial sample overlap unlikely.

**Figure 1:**
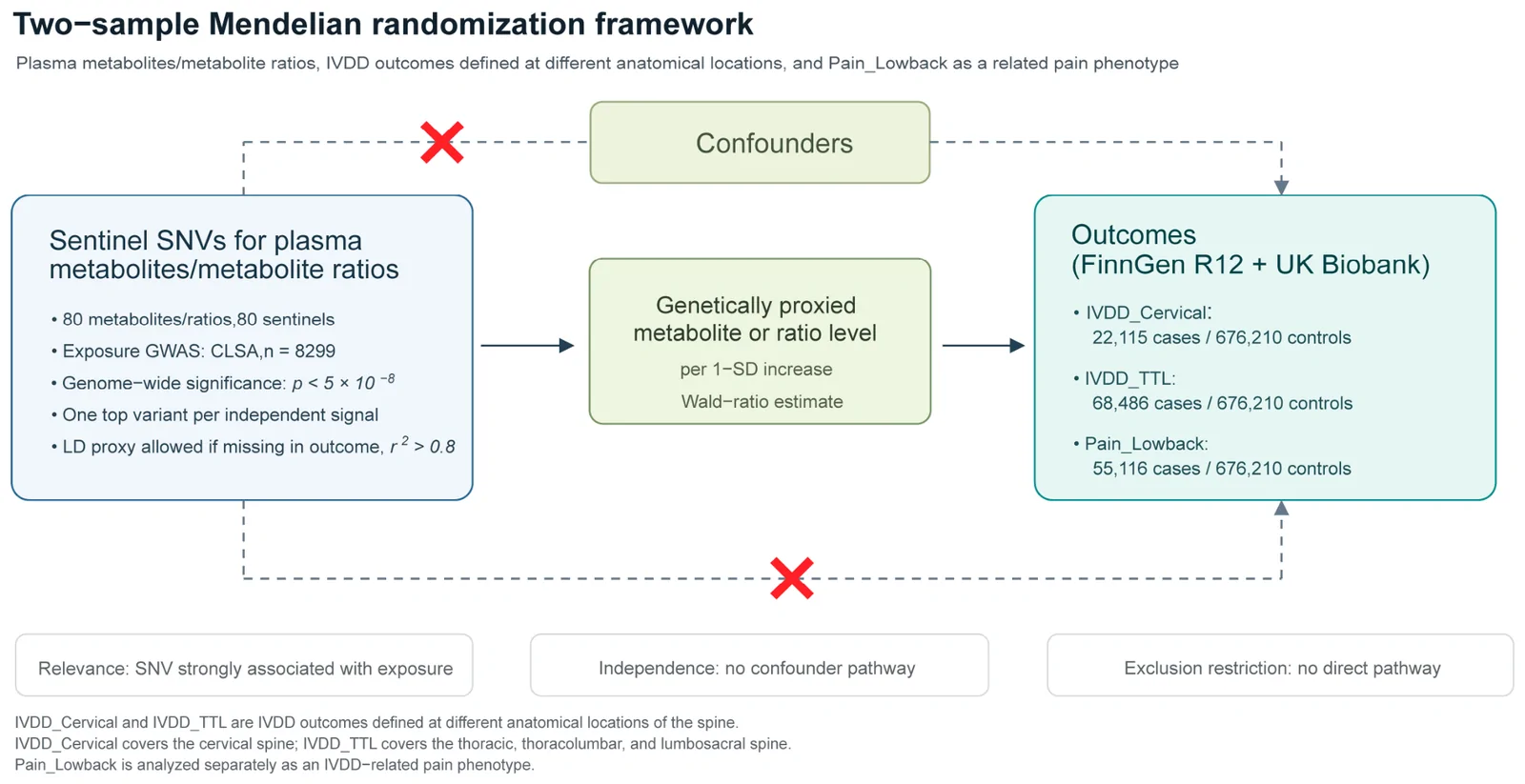
Two-sample Mendelian randomization framework for plasma metabolites and metabolite ratios, IVDD outcomes defined at different anatomical locations, and the related pain phenotype Pain_Lowback. Sentinel SNVs were selected from the European ancestry CLSA plasma metabolome GWAS at genome-wide significance, with one sentinel per exposure and an ancestry-matched LD proxy when necessary. IVDD_Cervical and IVDD_TTL represented IVDD outcomes defined at different anatomical locations of the spine, whereas Pain_Lowback was analysed separately as an IVDD-related pain phenotype. The three instrumental-variable assumptions are shown: relevance, independence, and exclusion restriction. CLSA, Canadian Longitudinal Study on Ageing; GWAS, genome-wide association study; IVDD, intervertebral disc degeneration; IVDD_Cervical, Intervertebral Disc Degeneration of Cervical Spine; IVDD_TTL, Intervertebral Disc Degeneration of Thoracic, Thoracolumbar, and Lumbosacral Spine; LD, linkage disequilibrium; MR, Mendelian randomization; Pain_Lowback, Low Back Pain; SD, standard deviation; SNV, single-nucleotide variant.

Each metabolite or metabolite ratio was treated as a separate exposure, and the three outcomes were analysed independently. Outcome associations were estimated on the log-odds scale and exponentiated to odds ratios (ORs). Instruments were required to satisfy the relevance, independence, and exclusion-restriction assumptions of MR [12], as summarised in Figure 1. Detailed GWAS sources are provided in Supplementary Table S1, and pharmacological annotations were obtained from DrugBank [13].

### 2.2. Instrument Selection and Data Harmonisation

Instrumental variables were selected using an exposure-side single-sentinel strategy. For each metabolite or ratio, the sentinel was the genome-wide significant single-nucleotide variant (SNV; *p* < 5 × 10^−8^) with the strongest exposure association and largest explained variance (R^2^) among independent signals assigned to the corresponding effector gene. The fatty acid desaturase (FADS) locus and the major histocompatibility complex (MHC) region on chromosome 6 were excluded to reduce broad pathway-level pleiotropy. When multiple independent signals were available, only the top sentinel was retained, yielding 80 exposures and 80 corresponding sentinel SNVs after filtering and outcome matching.

When a sentinel was unavailable in an outcome dataset, an ancestry-matched linkage disequilibrium (LD) proxy with r^2^ > 0.8 was permitted; the proxy with the highest r^2^ was selected when multiple candidates were available. Exposure and outcome associations were harmonised to the same effect allele, with effect signs reversed when required. Palindromic variants were retained only when allele-frequency information allowed unambiguous strand resolution; unresolved strand conflicts, non-A/T/C/G alleles, and records lacking required association fields were excluded. Because independence was enforced during sentinel selection, no additional LD clumping was performed at the MR stage.

### 2.3. MR Estimation, Pleiotropy Control, and Statistical Inference

Because each exposure contributed one sentinel instrument, the primary estimator was the Wald ratio implemented in TwoSampleMR. Estimates for binary outcomes were reported as ORs with 95% confidence intervals (CIs) per 1-standard-deviation (SD) increase in the genetically proxied metabolite or metabolite-ratio level. Instrument strength was assessed from the exposure-side variance explained (R^2^) and the first-stage F statistic calculated as

$$F=\frac{R^{2}/k}{\left(1-R^{2}\right)\left(n-k-1\right)}$$

where k = 1 and *n* is the exposure GWAS sample size. R^2^ and F statistics were reported for each instrument and were not used for additional filtering beyond the exposure-side genome-wide significance criterion. Post hoc power was summarised for prespecified odds ratios of 0.90, 1.20, and 1.50 using the case–control ratio of each outcome.

Potential horizontal pleiotropy was evaluated by screening each sentinel or selected proxy against the GWAS Catalogue with gwasrapidd (version 0.99.18) in June 2025. Genome-wide significant associations with musculoskeletal, inflammatory or immune, obesity-related, bone-metabolism, or major systemic traits were flagged and included in a prespecified drop list. Variants associated at genome-wide significance with the target outcome were also excluded before estimation. Cross-references for all evaluated instruments are provided in Supplementary Table S2.

All *p* values were two-sided. Multiple comparisons were controlled using Bonferroni correction within each outcome (0.05/80 = 6.25 × 10^−4^). Associations with *p* < 6.25 × 10^−4^ were defined as significant; associations with 6.25 × 10^−4^ ≤ *p* < 0.05 were defined as suggestive significant and treated as exploratory.

Cross-outcome consistency was examined descriptively across the anatomically defined IVDD outcomes and Pain_Lowback among associations with *p* < 0.05, including significant and suggestive significant results. Signals were indexed by metabolite–sentinel SNV pair, deduplicated across outcomes, and summarised according to their presence and Wald ratio direction in an UpSet plot and directional matrix. This descriptive comparison did not change the outcome-specific significance rule. Because the outcome datasets came from the same FinnGen R12 and UK Biobank meta-analysis and shared a common control set, overlap was treated as directional consistency across outcome definitions rather than independent replication.

Analyses were performed in R version 4.5.1. TwoSampleMR version 0.6.22 was used for data formatting, harmonisation, and Wald ratio estimation, and MendelianRandomization version 0.10.0 provided complementary utilities. Data management and figure production used data.table, dplyr, tidyr, reshape2, ggplot2, forestplot, plotly, htmlwidgets, and webshot. The scripted workflow generated harmonised datasets, MR estimates, instrument-strength summaries, figures, and analysis tables.

## 3. Results

### 3.1. Metabolite-Associated SNVs

Of 171 initially selected metabolite-associated SNVs, 103 remained after removal of non-canonical variants, and 100 were available in the outcome GWAS datasets by rsID matching. A further 20 SNVs with potentially confounding phenotype associations were excluded, leaving 80 strongly associated sentinel instruments for MR analysis (Supplementary Table S3). All instruments exceeded the conventional F-statistic threshold of 10 (range, 36.5–4013; Supplementary Table S4), indicating limited susceptibility to weak-instrument bias.

### 3.2. Single-Sentinel MR Associations Across IVDD_Cervical, IVDD_TTL, and Pain_Lowback

Eighty plasma metabolites and metabolite ratios were evaluated for each outcome using single-sentinel Wald ratio estimates.

Two associations were significant, both for IVDD_TTL. Higher genetically proxied dimethylarginine (SDMA + ADMA) was associated with lower IVDD_TTL risk (OR = 0.80; 95% CI: 0.72–0.90; *p* = 2.5 × 10^−4^), whereas higher alpha-hydroxyisovalerate was associated with higher risk (OR = 1.08; 95% CI: 1.04–1.13; *p* = 4.6 × 10^−4^). Five additional IVDD_TTL associations met the suggestive significant criterion.

No significant association was identified for IVDD_Cervical or Pain_Lowback. Ten IVDD_Cervical associations met the suggestive significant criterion, including dimethylglycine (OR = 1.10; 95% CI: 1.04–1.17; *p* = 0.002), N-formylmethionine (OR = 1.19; 95% CI: 1.06–1.34; *p* = 0.003), and alpha-hydroxyisovalerate (OR = 1.10; 95% CI: 1.02–1.19; *p* = 0.009). Eight associations with Pain_Lowback met the suggestive significant criterion, including 1,5-anhydroglucitol (1,5-AG) (OR = 1.19; 95% CI: 1.07–1.33; *p* = 0.001), alpha-hydroxyisovalerate (OR = 1.07; 95% CI: 1.02–1.12; *p* = 0.005), and S-1-pyrroline-5-carboxylate (OR = 0.96; 95% CI: 0.92–0.99; *p* = 0.008) (Supplementary Table S5).

**Figure 2:**
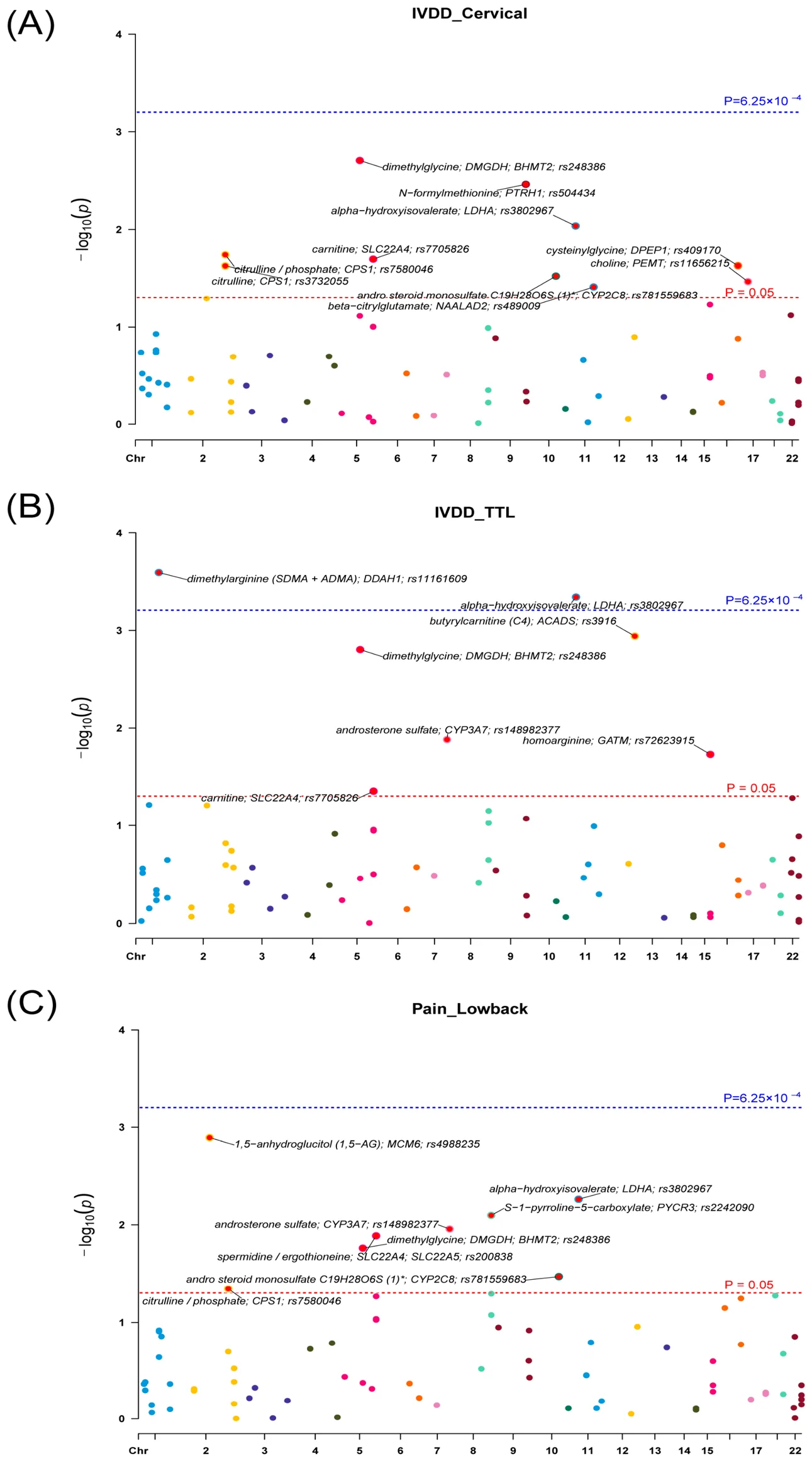
Single-sentinel Mendelian randomization scans of plasma metabolites across IVDD_Cervical, IVDD_TTL, and Pain_Lowback. (**A**) IVDD_Cervical, (**B**) IVDD_TTL, and (**C**) Pain_Lowback. The x-axis places the sentinel by chromosome, and the y-axis shows −log_10_(P). The red dashed line indicates *p* = 0.05, and the blue dashed line indicates the within-outcome Bonferroni threshold of *p* = 6.25 × 10^−4^. Each point represents one metabolite–outcome test using the sentinel SNV. Labels mark the top signals (metabolite; annotated gene; sentinel rsID). An asterisk indicates that metabolite unit: 1 s.d. of log-normalized values. IVDD_Cervical, Intervertebral Disc Degeneration of Cervical Spine; IVDD_TTL, Intervertebral Disc Degeneration of Thoracic, Thoracolumbar, and Lumbosacral Spine; MR, Mendelian randomization; Pain_Lowback, Low Back Pain; SNV, single-nucleotide variant.

**Figure 3:**
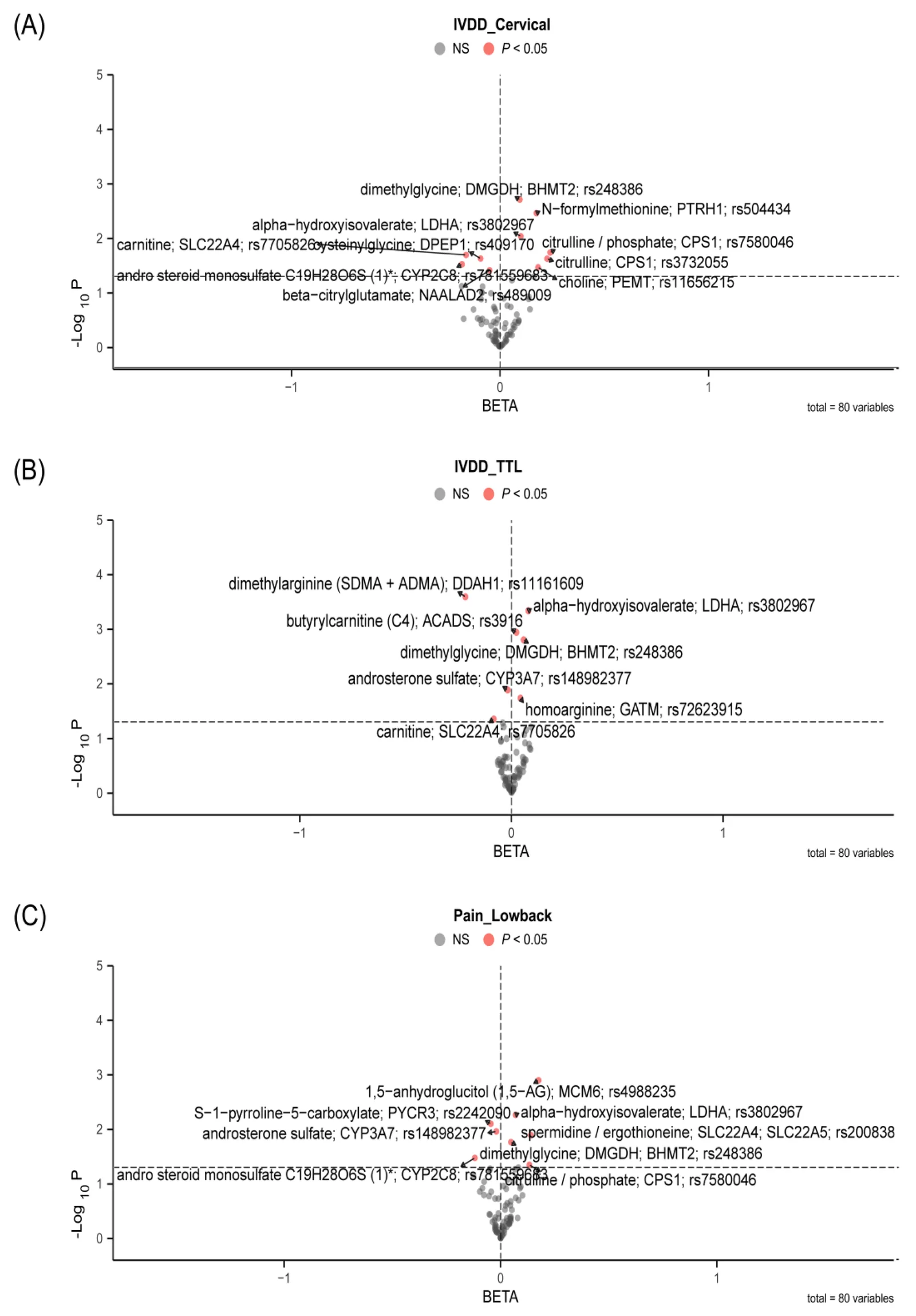
Single-sentinel Mendelian randomization volcano plots of plasma metabolites across IVDD_Cervical, IVDD_TTL, and Pain_Lowback. (**A**) IVDD_Cervical, (**B**) IVDD_TTL, and (**C**) Pain_Lowback. The x-axis is the MR β (log-odds per 1-SD higher genetically proxied metabolite level): β > 0 indicates higher risk (risk-increasing), β < 0 indicates lower risk (risk-decreasing). The y-axis is “−log_10_(P)”. The vertical dashed line marks “β = 0”; the horizontal dashed line marks the *p* = 0.05 suggestive threshold. The horizontal dashed line indicates *p* = 0.05, and the vertical dashed line indicates a null beta estimate. Each point represents one metabolite–outcome test using the sentinel SNV. Labels identify associations with *p* < 0.05 (metabolite; annotated gene; sentinel rsID). An asterisk indicates that metabolite unit: 1 s.d. of log-normalized values. 1,5-AG, 1,5-anhydroglucitol; ADMA, asymmetric dimethylarginine; IVDD_Cervical, Intervertebral Disc Degeneration of Cervical Spine; IVDD_TTL, Intervertebral Disc Degeneration of Thoracic, Thoracolumbar, and Lumbosacral Spine; NS, non-significant; Pain_Lowback, Low Back Pain; SDMA, symmetric dimethylarginine. All panels use the same −log10(P) y-axis range (0–5) to facilitate direct comparison; all observed data points are retained.

**Figure 4:**
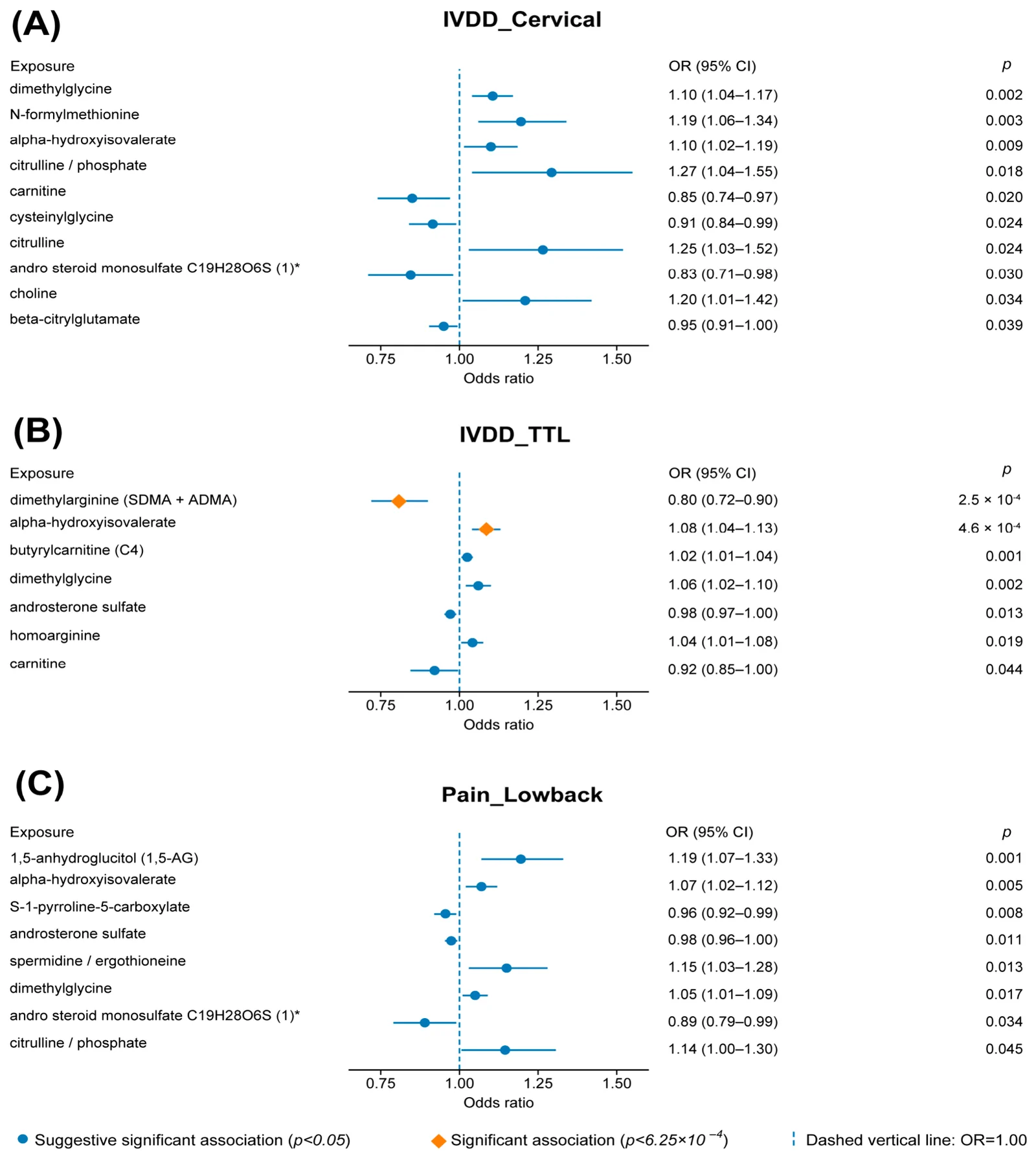
Focused forest plots of plasma metabolite associations with *p* < 0.05 across IVDD_Cervical, IVDD_TTL, and Pain_Lowback. (**A**) IVDD_Cervical (10 suggestive significant associations), (**B**) IVDD_TTL (two significant and five suggestive significant associations), and (**C**) Pain_Lowback (eight suggestive significant associations). Estimates are ORs with 95% CIs per 1-SD increase in the genetically proxied exposure. Orange diamonds denote significant associations; blue circles denote suggestive significant associations. An asterisk indicates that metabolite unit: 1 s.d. of log-normalized values. 1,5-AG, 1,5-anhydroglucitol; ADMA, asymmetric dimethylarginine; CI, confidence interval; IVDD_Cervical, Intervertebral Disc Degeneration of Cervical Spine; IVDD_TTL, Intervertebral Disc Degeneration of Thoracic, Thoracolumbar, and Lumbosacral Spine; OR, odds ratio; Pain_Lowback, Low Back Pain; SD, standard deviation; SDMA, symmetric dimethylarginine. The dashed vertical line denotes the null value (OR = 1.0), allowing the figure to be interpreted independently of the main text.

Figure 2 shows all 80 metabolite–outcome tests for each outcome, and Figure 3 displays their direction and statistical strength. Figure 4 presents the 25 associations with *p* < 0.05: 10 for IVDD_Cervical, seven for IVDD_TTL, and eight for Pain_Lowback. Complete forest plots for all 80 exposures are provided in Supplementary Figures S1–S3 for IVDD_Cervical, IVDD_TTL, and Pain_Lowback, respectively. Full numerical estimates are reported in Supplementary Tables S6–S8, and power summaries are provided in Supplementary Tables S9–S11.

### 3.3. Cross-Outcome Distribution of Metabolite Signals

Across the three outcomes, 25 metabolite–outcome associations had *p* < 0.05, including two significant and 23 suggestive significant associations. Deduplication by metabolite–sentinel SNV pair yielded 17 unique signals identified across the three outcomes. Six appeared in at least two outcomes, and 11 were outcome-specific. The outcome-level sets contained 10 signals for IVDD_Cervical, seven for IVDD_TTL, and eight for Pain_Lowback.

**Figure 5:**
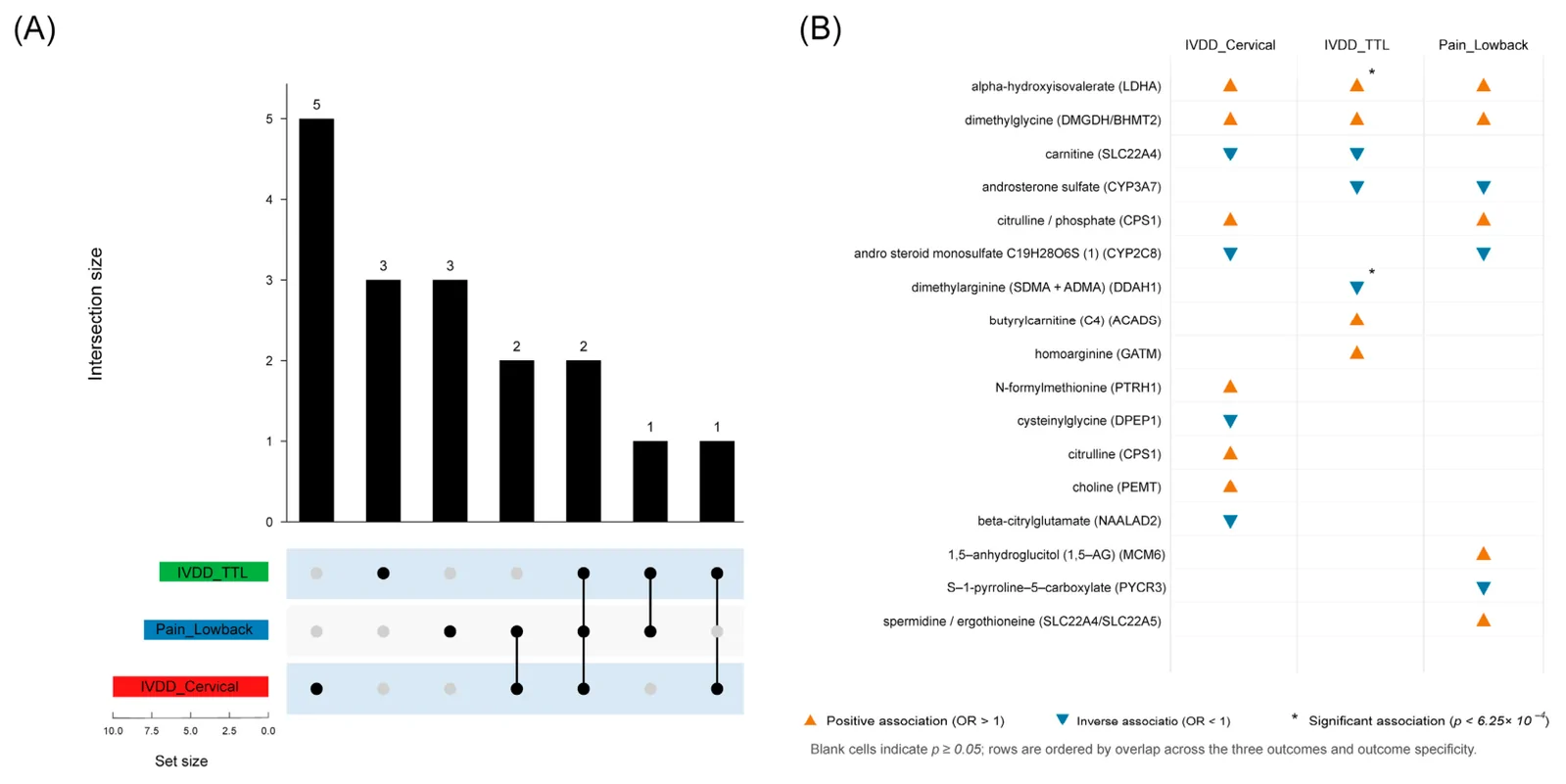
Cross-outcome distribution and direction of plasma metabolite signals with *p* < 0.05 across IVDD_Cervical, IVDD_TTL, and Pain_Lowback. (**A**) UpSet plot showing overlap of unique metabolite–sentinel SNV signals across IVDD_Cervical, IVDD_TTL, and Pain_Lowback. (**B**) Directional matrix showing positive associations (OR > 1), inverse associations (OR < 1), and significant results. Blank cells indicate *p* ≥ 0.05; rows are ordered by overlap across the three outcomes and outcome specificity. 1,5-AG, 1,5-anhydroglucitol; ADMA, asymmetric dimethylarginine; IVDD_Cervical, Intervertebral Disc Degeneration of Cervical Spine; IVDD_TTL, Intervertebral Disc Degeneration of Thoracic, Thoracolumbar, and Lumbosacral Spine; OR, odds ratio; Pain_Lowback, Low Back Pain; SDMA, symmetric dimethylarginine; SNV, single-nucleotide variant. In panel B, orange upward triangles indicate positive associations (OR > 1), blue downward triangles indicate inverse associations (OR < 1), and asterisks mark significant results.

Alpha-hydroxyisovalerate (LDHA) and dimethylglycine (DMGDH/BHMT2) were present in all three outcomes with concordant positive estimates. Carnitine, androsterone sulfate, citrulline/phosphate, and andro steroid monosulfate C19H28O6S (1) were each shared by two outcomes and had concordant directions. The remaining signals were specific to IVDD_Cervical (*n* = 5), IVDD_TTL (*n* = 3), or Pain_Lowback (*n* = 3). Only the IVDD_TTL associations with dimethylarginine (SDMA + ADMA) and alpha-hydroxyisovalerate were significant (Figure 5; Supplementary Table S12).

### 3.4. Functional and Pharmacological Annotation of Metabolite Signals

Functional annotation covered 16 effector genes mapped to the 23 suggestive significant metabolite–outcome associations. DrugBank mapping identified 487 unique drugs and bioactive compounds across 602 unique effector gene–compound pairs (Supplementary Table S13) [13].

Table 1 summarises compounds interacting with DDAH1 and LDHA, the effector enzymes assigned to the two significant IVDD_TTL signals. DDAH1 was linked to dexlansoprazole, pantoprazole, citrulline, and esomeprazole; dexlansoprazole and pantoprazole were annotated as inhibitors. LDHA was linked to artenimol, copper, etheno-NAD, NADH, nedosiran, nicotinamide, oxamic acid, and stiripentol; stiripentol was annotated as an inhibitor. These entries define the pharmacological context examined in the subsequent discussion.

## 4. Discussion

Using a uniform single-sentinel design, we examined 80 plasma metabolites and metabolite ratios across the anatomically defined IVDD outcomes and Pain_Lowback as a related pain phenotype. Two associations were significant, both for IVDD_TTL. Dimethylarginine (SDMA + ADMA), mapped to DDAH1, showed an inverse association, whereas alpha-hydroxyisovalerate, mapped to LDHA, showed a positive association. No significant association was identified for IVDD_Cervical or Pain_Lowback. Cross-outcome comparison identified several signals with concordant directions, and DrugBank annotation linked the two principal findings to compounds with documented interactions involving DDAH1 or LDHA [13]. The findings raise two main biological questions. The first concerns methylarginine turnover, nitric oxide signalling, and endplate-dependent nutrient exchange. The second concerns glycolytic adaptation and the consequences of lactate accumulation in the disc microenvironment. These pathways are biologically distinct but both are relevant to the survival of cells in an avascular, hypoxic, and nutrient-limited tissue.

Previous MR studies have also linked circulating metabolites to disc disease and spinal pain. Ji et al. evaluated 249 plasma metabolites and reported 13 associations with IVDD using multi-instrument analyses [14]. Jiang et al. examined 1091 metabolites and 309 metabolite ratios and identified associations involving amino-acid, fatty acid, nucleotide, and sphingolipid pathways, together with mediation analyses for selected micronutrients [15]. Wu et al. assessed 452 metabolites in relation to neck, thoracic, low back, and broader back pain phenotypes using bidirectional MR [16]. The limited overlap among reported metabolites probably reflects differences in metabolomic platforms, instrument availability, phenotype definitions, estimators, and multiple-testing procedures. It may also reflect the fact that metabolite loci often represent enzymes or transporters with broad biochemical effects, so apparently different measured traits can point to related metabolic processes. Our analysis complements the earlier work by applying the same single-sentinel rule to each exposure, separating the anatomically defined IVDD outcomes from the related pain outcome, and comparing the direction of shared signals across outcome definitions. The effector-gene and DrugBank annotations add biological context.

The concentration of significant findings in IVDD_TTL may reflect both statistical precision and phenotype biology. IVDD_TTL had the largest case count, which reduced uncertainty around its estimates, yet sample size alone is unlikely to explain the different leading signals. Cervical and thoracic, thoracolumbar, and lumbosacral discs experience different loading patterns, ranges of motion, endplate characteristics, and local vascular environments. Diagnostic coding may introduce additional variation because the IVDD_TTL outcome combines several anatomical regions and clinical presentations. Population data have reported region-specific associations between metabolic-syndrome components and disc degeneration, including comparatively clear findings in the thoracic region [17]. Such observations support metabolic heterogeneity across the spine, although they do not identify the pathway responsible for the present associations. Low back pain is even more heterogeneous. Disc, endplate, facet, muscular, neural, inflammatory, and central mechanisms can produce similar symptoms, and common lumbar MRI findings show only small associations with current or future pain severity in population cohorts [18]. A metabolic trait that is more closely related to structural disc pathology may therefore be diluted in a broad symptom phenotype. The concordant directions seen across the three outcomes remain useful for identifying shared biology, but the common control set means they should be viewed as consistency across definitions rather than separate replications.

The inverse IVDD_TTL estimate for dimethylarginine identifies a DDAH1-linked methylarginine signal. The exposure combines SDMA and ADMA, so the contribution of either component cannot be separated from the available summary statistics. This distinction is important because DDAH1 is an established enzyme in ADMA hydrolysis, whereas SDMA has different metabolic handling. ADMA inhibits nitric oxide synthase, and experimental and vascular studies connect the DDAH1–ADMA pathway with nitric oxide bioavailability, endothelial function, and vascular tone [19,20]. These processes are relevant to disc nutrition. The adult intervertebral disc has limited direct vascular supply, and solutes reach the nucleus pulposus primarily through diffusion across the vertebral endplate. Experimental reduction in endplate perfusion markedly decreases intradiscal transport [21], while degeneration is associated with endplate remodelling, reduced vascularity, and impaired small-molecule exchange [22]. The genetic signal may therefore reflect variation in methylarginine turnover and endothelial–endplate function that affects oxygen delivery, nutrient diffusion, and waste removal. This mechanism could operate systemically through vascular function or locally at the endplate microcirculation. Future studies should measure ADMA and SDMA separately, together with arginine, citrulline, nitric oxide metabolites, and indices of endplate perfusion. Colocalization and fine-mapping would help determine whether the metabolite and IVDD_TTL associations arise from the same causal variant and whether DDAH1 is the relevant effector gene.

The alpha-hydroxyisovalerate signal was annotated to LDHA, although the intermediate biochemical mechanism remains unresolved. Alpha-hydroxyisovalerate is a branched-chain amino-acid-related metabolite and is not itself lactate; the gene annotation therefore points to a candidate metabolic node rather than a demonstrated substrate–product relationship. Nevertheless, the link is consistent with evidence that glycolytic regulation is central to disc-cell homeostasis. Nucleus pulposus cells occupy a hypoxic and nutrient-limited environment and rely heavily on glycolysis for ATP generation. Experimental disruption of LDHA reduces glycolytic ATP production and accelerates degenerative cellular changes [23], while HIF-1α/PDK-1 signalling helps maintain glycolysis and mitochondrial integrity during oxidative stress [24]. These observations indicate that basal glycolytic activity is an adaptive requirement rather than a uniformly harmful process. The consequences change when glycolytic flux exceeds clearance capacity. Degenerated discs show increased lactate accumulation and lactylation, while experimental modulation of glycolysis and lactylation affects nucleus pulposus-cell senescence, autophagy, and matrix regulation [25]. Recent work has additionally linked glycolysis-derived lactate to H3K18-dependent ACSL4 transcription, ACSL4 lactylation, ferroptosis, and matrix dysfunction [26,27]. Acidification can amplify catabolic and pain-related responses through pathways that include ASIC3 [28]. The association is therefore compatible with a biphasic model in which physiological glycolysis supports survival, whereas sustained lactate accumulation, low pH, lactylation, and ferroptotic stress contribute to degeneration. This model favours experiments that quantify pathway state, metabolite flux, and tissue pH rather than assuming that general inhibition of LDHA would be beneficial.

The cross-outcome comparison identified six signals that appeared in at least two outcomes, all with concordant directions. Dimethylglycine, mapped to DMGDH/BHMT2, was the only non-Bonferroni signal present in all three outcomes. DMGDH and BHMT2 participate in the handling of dimethylglycine, betaine-related methyl groups, and one-carbon metabolism, which links amino-acid metabolism to redox balance and methyl-donor availability. Untargeted serum metabolomics in IVDD has identified changes in glycine-, serine-, threonine-, methionine-, and tricarboxylic-acid-related pathways [29]. These observations provide direct IVDD-specific pathway context, while broader evidence links one-carbon metabolism to redox balance and methyl-donor availability [30]. Neither line of evidence specifically establishes dimethylglycine as a causal mediator in disc tissue. Carnitine showed inverse associations with IVDD_Cervical and IVDD_TTL, whereas butyrylcarnitine showed a positive IVDD_TTL estimate. This pattern points to mitochondrial fatty acid transport and acylcarnitine handling [31]. Free carnitine availability, acyl-group buffering, and incomplete beta-oxidation can generate different circulating signatures, so the directions of carnitine and butyrylcarnitine need not be contradictory. Experimental correction of fatty acid metabolic dysfunction reduces nucleus pulposus-cell pyroptosis [32], and activation of PPARα–CPT1A-mediated lipid utilisation reduces lipid accumulation, senescence, and matrix degradation [33]. These findings make one-carbon metabolism and fatty acid utilisation reasonable secondary pathways for follow-up. Other shared signals, including steroid sulfates and citrulline/phosphate, currently have less direct disc-specific evidence and are better retained as hypothesis-generating observations.

DrugBank identified compounds with documented interactions involving DDAH1 or LDHA. The DDAH1 entries included dexlansoprazole and pantoprazole as annotated inhibitors, whereas the LDHA entries included stiripentol and the RNA-interference therapeutic nedosiran. Proton pump inhibitors have been examined in relation to DDAH1 biology, with experimental and structural studies showing that certain PPIs can inhibit or directly interact with DDAH1 [34,35]. Stiripentol has LDH-inhibitory activity [36], and nedosiran is an RNA-interference therapy designed to target hepatic LDHA expression in primary hyperoxaluria [37,38]. These examples show that both enzyme-linked nodes can be modified pharmacologically in other settings. Their relevance to IVDD, however, depends on tissue exposure, target engagement, dose, treatment duration, and the direction of the underlying biology. A liver-directed RNA-interference therapy, for example, cannot be assumed to reproduce genetic or cellular effects in the disc. DrugBank mapping is most useful here as a means of identifying existing chemical and therapeutic tools for experiments. Follow-up studies could test whether these compounds alter ADMA turnover, glycolytic flux, lactate production, pH, or matrix homeostasis in disc-relevant cells, while pharmacoepidemiological analyses could examine whether long-term exposure is associated with spinal outcomes.

The study has several strengths. A uniform genome-wide significance threshold and single-sentinel rule were applied to all exposures, instrument strength was high, and IVDD_Cervical, IVDD_TTL, and Pain_Lowback were analysed separately using prespecified significance criteria. Screening of catalogued variant associations and exclusion of broad pleiotropic regions reduced some obvious alternative pathways. The cross-outcome comparison and effector-gene annotation also helped connect statistical associations with specific biochemical questions. Several limitations affect interpretation. One instrument per exposure precluded MR-Egger, weighted-median estimation, Cochran’s Q, MR-PRESSO, and leave-one-out analyses, leaving residual horizontal pleiotropy unresolved. The GWAS Catalogue screen can identify known associations but cannot exclude unreported or tissue-specific pleiotropic effects. Effector-gene annotation requires confirmation because proximity or biochemical assignment does not prove that the annotated gene mediates the metabolite–outcome association. Shared controls may increase apparent agreement across outcomes, and registry-based definitions may include phenotype misclassification. Circulating metabolite levels may also differ from concentrations within the avascular disc and endplate microenvironment. The exposure and outcome GWAS were based predominantly on participants of European ancestry, which limits generalizability. Allele frequencies and linkage disequilibrium patterns can differ across ancestry groups, potentially altering instrument strength, proxy performance, and the transferability of genetically proxied associations; accordingly, these estimates should not be directly generalised to ethnically diverse global populations without external validation. Replication in well-powered cohorts of diverse ancestry is therefore needed. Age- and sex-specific effects could not be evaluated with the available summary-level data, and heterogeneity related to these factors may remain across source cohorts. The next priority is to determine whether the two IVDD_TTL signals can be reproduced and localised to causal variants at the loci of their annotated genes. Independent metabolite GWAS would provide external replication, while Steiger directionality testing, colocalization, fine-mapping, and integration with eQTL or sQTL data would clarify the genetic architecture. Targeted measurement of ADMA, SDMA, arginine, citrulline, alpha-hydroxyisovalerate, lactate, and acylcarnitines in plasma, vertebral endplate, and disc tissue could establish whether systemic genetic signals correspond to local metabolic states. Imaging studies incorporating endplate perfusion, Modic changes, and quantitative disc degeneration could connect the metabolic findings to structural phenotypes. Mechanistic work in nucleus pulposus, annulus fibrosus, and cartilage-endplate models should examine nutrient transport and nitric oxide signalling for the DDAH1-related signal, and glycolytic flux, lactate clearance, pH, lactylation, mitochondrial stress, and ferroptosis for the LDHA-related signal. Dimethylglycine and carnitine-related pathways can be evaluated in parallel as secondary candidates. A stepwise programme combining genetic replication, targeted metabolomics, imaging, and tissue-specific experiments would provide the clearest route from circulating associations to disc biology.

## 5. Conclusions

Among the 80 plasma metabolites and metabolite ratios evaluated for each outcome, two associations were significant, both for IVDD_TTL. Dimethylarginine (SDMA + ADMA), mapped to DDAH1, showed an inverse association, whereas alpha-hydroxyisovalerate, mapped to LDHA, showed a positive association. The results highlight two candidate biological pathways for further study: methylarginine-related nitric oxide and endplate physiology, and glycolytic and lactate-related stress in disc tissue.

No significant association was identified for IVDD_Cervical or Pain_Lowback. Shared cross-outcome patterns, including dimethylglycine and carnitine-related signals, remain exploratory because the outcome datasets used a common control set. Independent replication, colocalization or fine-mapping, targeted metabolomics, and disc-relevant experimental studies are needed to clarify the underlying mediators and determine whether these systemic genetic signals correspond to local metabolic changes in IVDD.

## Figures and Tables

**Table 1:** DrugBank-annotated compounds interacting with effector-gene products DDAH1 and LDHA for significant IVDD_TTL-associated metabolites.

Metabolite or Ratio	Effector Gene	Protein Type	DrugBank ID	Drug Name	Drug Group	Relation	Pharm. Action	DrugBank Action	Antag.	Agon.	Substr.	Inhib.	Induc.
dimethylarginine (SDMA + ADMA)	DDAH1	Protein	DB00155	Citrulline	Investigational; Nutraceutical	Target	Unknown	Not specified	0	0	0	0	0
dimethylarginine (SDMA + ADMA)	DDAH1	Protein	DB05351	Dexlansoprazole	Approved; Investigational	Target	No	Inhibitor	0	0	0	1	0
dimethylarginine (SDMA + ADMA)	DDAH1	Protein	DB00736	Esomeprazole	Approved; Investigational	Target	Unknown	Not specified	0	0	0	0	0
dimethylarginine (SDMA + ADMA)	DDAH1	Protein	DB00213	Pantoprazole	Approved; Investigational	Target	No	Inhibitor	0	0	0	1	0
alpha-hydroxyisovalerate	LDHA	Protein	DB11638	Artenimol	Approved; Investigational	Target	Unknown	Ligand	0	0	0	0	0
alpha-hydroxyisovalerate	LDHA	Protein	DB09130	Copper	Approved; Investigational	Target	Unknown	Not specified	0	0	0	0	0
alpha-hydroxyisovalerate	LDHA	Protein	DB02483	Etheno-NAD	Experimental	Target	Unknown	Not specified	0	0	0	0	0
alpha-hydroxyisovalerate	LDHA	Protein	DB00157	NADH	Approved; Nutraceutical	Target	Unknown	Not specified	0	0	0	0	0
alpha-hydroxyisovalerate	LDHA	Protein	DB17635	Nedosiran	Approved; Investigational	Target	Yes	siRNA (RNA interference)	0	0	0	0	0
alpha-hydroxyisovalerate	LDHA	Protein	DB02701	Nicotinamide	Approved; Investigational	Target	Unknown	Not specified	0	0	0	0	0
alpha-hydroxyisovalerate	LDHA	Protein	DB03940	Oxamic Acid	Experimental	Target	Unknown	Not specified	0	0	0	0	0
alpha-hydroxyisovalerate	LDHA	Protein	DB09118	Stiripentol	Approved; Investigational	Target	Yes	Inhibitor	0	0	0	1	0

Note: The table links the significant single-sentinel MR associations for IVDD_TTL—dimethylarginine (SDMA + ADMA) and alpha-hydroxyisovalerate—to their assigned effector genes DDAH1 and LDHA and lists DrugBank-annotated compounds. Drug names, DrugBank identifiers, relation types, action annotations, and binary action fields are unchanged from the source data. Binary action columns—Antag. (antagonist), Agon. (agonist), Substr. (substrate), Inhib. (inhibitor), and Induc. (inducer)—indicate whether the corresponding DrugBank action is annotated (1) or not annotated (0); ‘Unknown’ and ‘Not specified’ reproduce the source annotation. ADMA, asymmetric dimethylarginine; DDAH1, dimethylarginine dimethylaminohydrolase 1; IVDD_TTL, Intervertebral Disc Degeneration of Thoracic, Thoracolumbar, and Lumbosacral Spine; LDHA, lactate dehydrogenase A; MR, Mendelian randomization; NAD, nicotinamide adenine dinucleotide; NADH, reduced nicotinamide adenine dinucleotide; SDMA, symmetric dimethylarginine; siRNA, small-interfering RNA.

## Data Availability

The data presented in this study are available in the article and Supplementary Materials. Public summary statistics for the IVDD outcomes defined at different anatomical locations (IVDD_Cervical and IVDD_TTL) and the related pain phenotype Pain_Lowback were obtained from the FinnGen R12 resource and the UK Biobank-based meta-analysis. Plasma metabolite and metabolite-ratio summary statistics were derived from the Canadian Longitudinal Study on Ageing (CLSA) cohort and accessed through the GWAS Catalogue for European ancestry under accession ranges GCST90199621–GCST90201020. Variant cross-references were retrieved from the GWAS Catalogue, and pharmacological annotations linking metabolites, effector genes, and drugs were obtained from DrugBank.

## Supplementary Materials

The following supporting information can be downloaded at https://www.mdpi.com/article/10.3390/metabo16090673/s1.

## Abbreviations

1,5-AG, 1,5-anhydroglucitol; ACSL4, acyl-CoA synthetase long-chain family member 4; ADMA, asymmetric dimethylarginine; ASIC3, acid-sensing ion channel 3; BHMT2, betaine-homocysteine S-methyltransferase 2; CI, confidence interval; CLSA, Canadian Longitudinal Study on Ageing; CPT1A, carnitine palmitoyltransferase 1A; DDAH1, dimethylarginine dimethylaminohydrolase 1; DMGDH, dimethylglycine dehydrogenase; eQTL, expression quantitative trait locus; FADS, fatty acid desaturase; GWAS, genome-wide association study; HIF-1α, hypoxia-inducible factor 1-alpha; IV, instrumental variable; IVDD, intervertebral disc degeneration; IVDD_Cervical, Intervertebral Disc Degeneration of Cervical Spine; IVDD_TTL, Intervertebral Disc Degeneration of Thoracic, Thoracolumbar, and Lumbosacral Spine; LD, linkage disequilibrium; LDHA, lactate dehydrogenase A; MHC, major histocompatibility complex; MR, Mendelian randomization; MR-Egger, Mendelian randomization Egger regression; MR-PRESSO, Mendelian Randomization Pleiotropy RESidual Sum and Outlier; NAD, nicotinamide adenine dinucleotide; NADH, reduced nicotinamide adenine dinucleotide; NO, nitric oxide; OR, odds ratio; Pain_Lowback, Low Back Pain; PDK-1, pyruvate dehydrogenase kinase 1; PPARα, peroxisome proliferator-activated receptor alpha; PPI, proton pump inhibitor; QTL, quantitative trait locus; RCT, randomised controlled trial; SD, standard deviation; SDMA, symmetric dimethylarginine; siRNA, small-interfering RNA; SNV, single-nucleotide variant; sQTL, splicing quantitative trait locus; STROBE-MR, Strengthening the Reporting of Observational Studies in Epidemiology using Mendelian Randomization.
