# Supplementary material for: Causal Effects of Plasma Metabolites on Intervertebral Disc Degeneration and Low Back Pain: A Mendelian Randomization Study
Source: Metabolites. 2026 Sep 13;16(9):673. doi: 10.3390/metabo16090673 (PMC13609130; doi:10.3390/metabo16090673)
Supplement: Supplementary file 1 [file metabolites-16-00673-s001.zip › metabolites-4511828-supplementary figures.pdf]

SUPPLEMENTARY FIGURE S1

IVDD_Cervical

Exposures	N−SNV	OR (95% CI)	P
dimethylglycine	1	1.10 (1.04, 1.17)	0.002
N−formylmethionine	1	1.19 (1.06, 1.34)	0.003
alpha−hydroxyisovalerate	1	1.10 (1.02, 1.19)	0.009
citrulline / phosphate	1	1.27 (1.04, 1.55)	0.018
carnitine	1	0.85 (0.74, 0.97)	0.02
cysteinylglycine	1	0.91 (0.84, 0.99)	0.024
citrulline	1	1.25 (1.03, 1.52)	0.024
andro steroid monosulfate C19H28O6S (1)*	1	0.83 (0.71, 0.98)	0.03
choline	1	1.20 (1.01, 1.42)	0.034
beta−citrylglutamate	1	0.95 (0.91, 1.00)	0.039
1,5−anhydroglucitol (1,5−AG)	1	1.18 (1.00, 1.38)	0.051
homoarginine	1	1.06 (1.00, 1.12)	0.059
3−methoxytyrosine	1	0.83 (0.68, 1.02)	0.076
betaine	1	0.92 (0.84, 1.01)	0.077
carnitine / ergothioneine	1	0.92 (0.83, 1.02)	0.099
homostachydrine*	1	0.92 (0.83, 1.02)	0.1
S−1−pyrroline−5−carboxylate	1	0.96 (0.91, 1.01)	0.102
caprylate (8:0)	1	1.15 (0.97, 1.36)	0.118
butyrylcarnitine (C4)	1	1.02 (0.99, 1.04)	0.127
glycine	1	1.15 (0.96, 1.37)	0.13
cysteinylglycine / taurine	1	0.91 (0.81, 1.03)	0.132
hexanoylcarnitine (C6)	1	1.04 (0.98, 1.09)	0.173
hexanoylglycine	1	1.05 (0.98, 1.14)	0.182
4−guanidinobutanoate	1	0.97 (0.93, 1.01)	0.182
orotate	1	0.97 (0.94, 1.01)	0.197
tryptophan	1	1.15 (0.93, 1.44)	0.201
2−hydroxyglutarate	1	0.88 (0.72, 1.07)	0.202
N−acetyl−aspartyl−glutamate (NAAG)	1	0.98 (0.95, 1.01)	0.219
N−acetylglucosaminylasparagine	1	1.08 (0.95, 1.24)	0.251
sphingosine 1−phosphate	1	0.90 (0.73, 1.10)	0.295
phosphate / tyrosine	1	0.84 (0.60, 1.17)	0.301
uridine	1	0.92 (0.78, 1.08)	0.301
androsterone sulfate	1	0.99 (0.96, 1.01)	0.309
sphinganine−1−phosphate	1	0.91 (0.76, 1.09)	0.315
creatine / carnitine	1	1.09 (0.92, 1.31)	0.319
creatine	1	1.09 (0.91, 1.31)	0.333
N−acetylhistidine	1	0.97 (0.91, 1.03)	0.342
sebacate (C10−DC)	1	1.07 (0.93, 1.25)	0.343
2'−deoxyuridine / cytidine	1	0.94 (0.82, 1.07)	0.345
5,6−dihydrothymine	1	1.09 (0.91, 1.31)	0.356
5−methyluridine (ribothymidine)	1	0.98 (0.94, 1.02)	0.361
bilirubin (Z,Z) / etiocholanolone glucuronide	1	1.03 (0.97, 1.09)	0.365
dimethylarginine (SDMA + ADMA)	1	0.91 (0.75, 1.11)	0.374
glycine / serine	1	1.07 (0.92, 1.23)	0.392
beta−alanine	1	0.96 (0.87, 1.06)	0.401
phosphate / uridine	1	1.07 (0.90, 1.28)	0.427
phosphate / 5−oxoproline	1	1.02 (0.97, 1.08)	0.444
phenylpyruvate	1	1.06 (0.90, 1.26)	0.461
N−palmitoylglycine	1	0.98 (0.92, 1.04)	0.494
3beta−hydroxy−5−cholestenoate	1	0.98 (0.92, 1.04)	0.511
adenosine 3',5'−cyclic monophosphate (cAMP)	1	1.05 (0.90, 1.23)	0.522
gamma−CEHC	1	0.98 (0.91, 1.06)	0.574
citrulline / dimethylarginine (SDMA + ADMA)	1	1.05 (0.89, 1.24)	0.582
cholic acid glucuronide	1	0.98 (0.93, 1.04)	0.588
deoxycholic acid glucuronide	1	0.99 (0.97, 1.02)	0.588
thyroxine	1	0.95 (0.78, 1.15)	0.589
2'−deoxyuridine	1	1.04 (0.91, 1.18)	0.594
glutamate / 5−oxoproline	1	1.03 (0.91, 1.17)	0.595
myo−inositol	1	1.06 (0.86, 1.31)	0.598
O−sulfo−L−tyrosine	1	0.96 (0.81, 1.13)	0.632
serine	1	0.98 (0.88, 1.09)	0.667
N−acetylputrescine / (N(1) + N(8))−acetylspermidine	1	1.03 (0.90, 1.17)	0.692
cholesterol / cortisol	1	0.97 (0.80, 1.17)	0.737
erythronate*	1	0.98 (0.87, 1.11)	0.743
4−oxo−retinoic acid	1	1.03 (0.86, 1.22)	0.75
cortisol	1	1.03 (0.85, 1.25)	0.753
N−alpha−acetylornithine	1	0.98 (0.87, 1.11)	0.758
4−cholesten−3−one	1	0.97 (0.82, 1.16)	0.772
pregnenediol sulfate (C21H34O5S)*	1	0.97 (0.80, 1.18)	0.779
uridine / pseudouridine	1	1.02 (0.84, 1.24)	0.817
S−adenosylhomocysteine (SAH) / leucine	1	0.99 (0.88, 1.11)	0.825
hypotaurine	1	0.98 (0.80, 1.20)	0.849
hydantoin−5−propionate	1	1.01 (0.85, 1.21)	0.885
beta−hydroxyisovalerate	1	1.01 (0.86, 1.18)	0.914
lithocholate sulfate (1)	1	1.01 (0.90, 1.12)	0.915
cysteine−glutathione disulfide	1	1.00 (0.93, 1.07)	0.935
spermidine / ergothioneine	1	0.99 (0.84, 1.18)	0.942
N−acetylglycine	1	1.00 (0.84, 1.18)	0.956
succinimide	1	1.00 (0.86, 1.16)	0.976
3−formylindole	1	1.00 (0.91, 1.09)	0.977

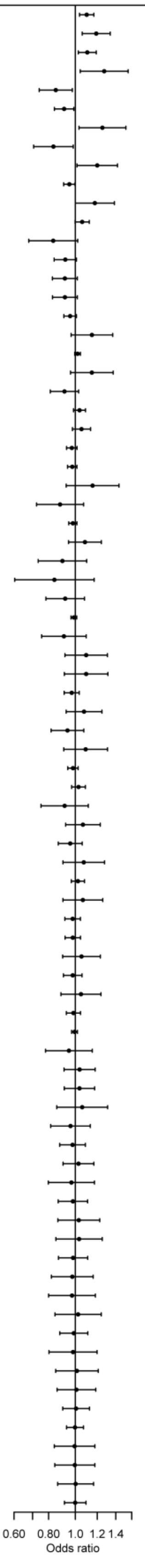

SUPPLEMENTARY FIGURE S1 Complete single-sentinel Mendelian randomization forest plot for IVDD_Cervical. IVDD_Cervical and IVDD_TTL are intervertebral disc degeneration outcomes defined at different anatomical locations of the spine; this figure presents the cervical-spine outcome. Each row represents one metabolite–outcome test using a single sentinel SNV and the Wald-ratio estimator. Points show ORs per 1-SD increase in genetically proxied metabolite or metabolite-ratio level, and horizontal bars show 95% CIs. The vertical dashed line marks the null value (OR = 1). The N-SNV column (= 1 for all rows) indicates that each estimate is based on a single instrument. An asterisk indicates transformed exposure units: 1 SD of log-normalized values for metabolites and 1 SD of inverse rank-normalized values for metabolite ratios. CI, confidence interval; IVDD, intervertebral disc degeneration; IVDD_Cervical, Intervertebral Disc Degeneration of Cervical Spine; IVDD_TTL, Intervertebral Disc Degeneration of Thoracic, Thoracolumbar, and Lumbosacral Spine; MR, Mendelian randomization; N-SNV, number of single-nucleotide variants; OR, odds ratio; SD, standard deviation; SNV, single-nucleotide variant.

SUPPLEMENTARY FIGURE S2

IVDD_TTL

Exposures	N−SNV	OR (95% CI)	P
dimethylarginine (SDMA + ADMA)	1	0.80 (0.72, 0.90)	0.00025
alpha−hydroxyisovalerate	1	1.08 (1.04, 1.13)	0.00046
butyrylcarnitine (C4)	1	1.02 (1.01, 1.04)	0.001
dimethylglycine	1	1.06 (1.02, 1.10)	0.002
androsterone sulfate	1	0.98 (0.97, 1.00)	0.013
homoarginine	1	1.04 (1.01, 1.08)	0.019
carnitine	1	0.92 (0.85, 1.00)	0.044
cysteine−glutathione disulfide	1	0.96 (0.92, 1.00)	0.052
N−palmitoylglycine	1	0.96 (0.93, 1.00)	0.061
1,5−anhydroglucitol (1,5−AG)	1	1.10 (1.00, 1.21)	0.062
glutamate / 5−oxoproline	1	1.07 (0.99, 1.15)	0.071
N−formylmethionine	1	1.06 (0.99, 1.14)	0.085
phosphate / 5−oxoproline	1	1.03 (1.00, 1.06)	0.094
beta−citrylglutamate	1	0.98 (0.95, 1.00)	0.102
carnitine / ergothioneine	1	0.95 (0.89, 1.01)	0.11
homostachydrine*	1	0.95 (0.89, 1.01)	0.113
N−acetylglucosaminylasparagine	1	1.07 (0.98, 1.16)	0.122
5,6−dihydrothymine	1	1.09 (0.98, 1.21)	0.129
citrulline / phosphate	1	1.09 (0.97, 1.23)	0.152
myo−inositol	1	1.09 (0.97, 1.24)	0.159
bilirubin (Z,Z) / etiocholanolone glucuronide	1	1.02 (0.99, 1.06)	0.181
3−formylindole	1	0.97 (0.92, 1.02)	0.22
gamma−CEHC	1	0.97 (0.93, 1.02)	0.222
S−1−pyrroline−5−carboxylate	1	0.98 (0.95, 1.01)	0.224
glycine / serine	1	1.05 (0.97, 1.15)	0.224
hydantoin−5−propionate	1	1.06 (0.96, 1.18)	0.245
N−acetylglycine	1	0.94 (0.85, 1.04)	0.248
citrulline	1	1.07 (0.95, 1.20)	0.252
S−adenosylhomocysteine (SAH) / leucine	1	0.96 (0.90, 1.03)	0.266
2−hydroxyglutarate	1	0.94 (0.83, 1.05)	0.267
erythronate*	1	0.96 (0.89, 1.03)	0.268
phosphate / uridine	1	1.06 (0.96, 1.17)	0.273
glycine	1	1.06 (0.95, 1.18)	0.286
3−methoxytyrosine	1	0.94 (0.83, 1.06)	0.302
uridine	1	0.95 (0.87, 1.05)	0.303
spermidine / ergothioneine	1	0.95 (0.86, 1.05)	0.313
uridine / pseudouridine	1	1.06 (0.94, 1.19)	0.324
2'−deoxyuridine / cytidine	1	0.96 (0.89, 1.04)	0.325
N−acetyl−aspartyl−glutamate (NAAG)	1	0.99 (0.97, 1.01)	0.341
betaine	1	0.97 (0.92, 1.03)	0.346
cysteinylglycine / taurine	1	1.03 (0.96, 1.11)	0.36
beta−alanine	1	1.03 (0.97, 1.09)	0.384
succinimide	1	1.04 (0.95, 1.14)	0.385
tryptophan	1	1.06 (0.93, 1.20)	0.407
sphinganine−1−phosphate	1	0.95 (0.86, 1.07)	0.409
sphingosine 1−phosphate	1	0.95 (0.84, 1.07)	0.414
caprylate (8:0)	1	1.04 (0.94, 1.15)	0.456
choline	1	1.04 (0.94, 1.15)	0.487
hexanoylcarnitine (C6)	1	1.01 (0.98, 1.04)	0.504
3beta−hydroxy−5−cholestenoate	1	1.01 (0.98, 1.05)	0.504
pregnenediol sulfate (C21H34O5S)*	1	0.96 (0.86, 1.08)	0.519
cysteinylglycine	1	1.02 (0.97, 1.07)	0.52
phenylpyruvate	1	1.03 (0.94, 1.14)	0.522
beta−hydroxyisovalerate	1	0.97 (0.88, 1.07)	0.534
5−methyluridine (ribothymidine)	1	0.99 (0.97, 1.02)	0.539
serine	1	0.98 (0.92, 1.04)	0.547
4−cholesten−3−one	1	0.97 (0.88, 1.08)	0.58
hexanoylglycine	1	1.01 (0.97, 1.06)	0.581
andro steroid monosulfate C19H28O6S (1)*	1	0.97 (0.88, 1.08)	0.594
thyroxine	1	0.98 (0.87, 1.09)	0.669
N−acetylhistidine	1	0.99 (0.96, 1.03)	0.686
sebacate (C10−DC)	1	1.02 (0.93, 1.11)	0.702
orotate	1	1.00 (0.97, 1.02)	0.708
phosphate / tyrosine	1	0.97 (0.82, 1.15)	0.715
4−oxo−retinoic acid	1	0.98 (0.89, 1.09)	0.746
lithocholate sulfate (1)	1	0.99 (0.93, 1.06)	0.786
creatine	1	0.99 (0.88, 1.10)	0.788
cholic acid glucuronide	1	1.00 (0.96, 1.03)	0.817
deoxycholic acid glucuronide	1	1.00 (0.98, 1.01)	0.817
cholesterol / cortisol	1	0.99 (0.88, 1.11)	0.821
citrulline / dimethylarginine (SDMA + ADMA)	1	1.01 (0.92, 1.12)	0.829
N−alpha−acetylornithine	1	0.99 (0.92, 1.07)	0.852
N−acetylputrescine / (N(1) + N(8))−acetylspermidine	1	0.99 (0.92, 1.07)	0.859
cortisol	1	1.01 (0.90, 1.13)	0.861
creatine / carnitine	1	0.99 (0.89, 1.10)	0.862
adenosine 3',5'−cyclic monophosphate (cAMP)	1	1.01 (0.92, 1.11)	0.874
O−sulfo−L−tyrosine	1	1.00 (0.91, 1.10)	0.918
4−guanidinobutanoate	1	1.00 (0.98, 1.03)	0.943
2'−deoxyuridine	1	1.00 (0.93, 1.08)	0.962
hypotaurine	1	1.00 (0.89, 1.13)	0.988

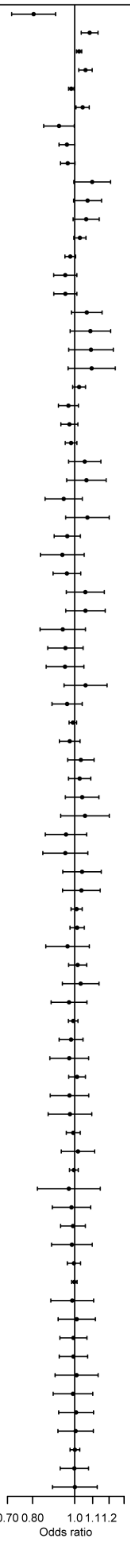

SUPPLEMENTARY FIGURE S2 Complete single-sentinel Mendelian randomization forest plot for IVDD_TTL. IVDD_Cervical and IVDD_TTL are intervertebral disc degeneration outcomes defined at different anatomical locations of the spine; this figure presents the thoracic, thoracolumbar, and lumbosacral-spine outcome. Each row represents one metabolite–outcome test using a single sentinel SNV and the Wald-ratio estimator. Points show ORs per 1-SD increase in genetically proxied metabolite or metabolite-ratio level, and horizontal bars show 95% CIs. The vertical dashed line marks the null value (OR = 1). The N-SNV column (= 1 for all rows) indicates that each estimate is based on a single instrument. An asterisk indicates transformed exposure units: 1 SD of log-normalized values for metabolites and 1 SD of inverse rank-normalized values for metabolite ratios. CI, confidence interval; IVDD, intervertebral disc degeneration; IVDD_Cervical, Intervertebral Disc Degeneration of Cervical Spine; IVDD_TTL, Intervertebral Disc Degeneration of Thoracic, Thoracolumbar, and Lumbosacral Spine; MR, Mendelian randomization; N-SNV, number of single-nucleotide variants; OR, odds ratio; SD, standard deviation; SNV, single-nucleotide variant.

SUPPLEMENTARY FIGURE S3

Pain_Lowback

Exposures	N−SNV	OR (95% CI)	P
1,5−anhydroglucitol (1,5−AG)	1	1.19 (1.07, 1.33)	0.001
alpha−hydroxyisovalerate	1	1.07 (1.02, 1.12)	0.005
S−1−pyrroline−5−carboxylate	1	0.96 (0.92, 0.99)	0.008
androsterone sulfate	1	0.98 (0.96, 1.00)	0.011
spermidine / ergothioneine	1	1.15 (1.03, 1.28)	0.013
dimethylglycine	1	1.05 (1.01, 1.09)	0.017
andro steroid monosulfate C19H28O6S (1)*	1	0.89 (0.79, 0.99)	0.034
citrulline / phosphate	1	1.14 (1.00, 1.30)	0.045
glutamate / 5−oxoproline	1	1.09 (1.00, 1.18)	0.051
gamma−CEHC	1	0.95 (0.91, 1.00)	0.053
carnitine / ergothioneine	1	1.07 (1.00, 1.14)	0.054
cysteinylglycine	1	0.95 (0.90, 1.00)	0.057
myo−inositol	1	0.88 (0.77, 1.01)	0.071
phosphate / 5−oxoproline	1	1.03 (1.00, 1.07)	0.084
carnitine	1	0.93 (0.85, 1.01)	0.092
homostachydrine*	1	1.06 (0.99, 1.13)	0.094
butyrylcarnitine (C4)	1	1.01 (1.00, 1.03)	0.111
glycine	1	1.10 (0.98, 1.23)	0.113
caprylate (8:0)	1	1.09 (0.98, 1.22)	0.121
phenylpyruvate	1	0.92 (0.83, 1.02)	0.121
hexanoylcarnitine (C6)	1	1.03 (0.99, 1.06)	0.124
dimethylarginine (SDMA + ADMA)	1	0.91 (0.80, 1.03)	0.139
3−formylindole	1	0.96 (0.90, 1.01)	0.14
N−acetylglycine	1	0.93 (0.83, 1.03)	0.161
tryptophan	1	1.11 (0.96, 1.27)	0.163
cysteinylglycine / taurine	1	0.95 (0.88, 1.02)	0.169
adenosine 3',5'−cyclic monophosphate (cAMP)	1	1.07 (0.97, 1.19)	0.182
cholic acid glucuronide	1	0.97 (0.94, 1.01)	0.187
deoxycholic acid glucuronide	1	0.99 (0.97, 1.01)	0.188
citrulline	1	1.09 (0.96, 1.23)	0.2
lithocholate sulfate (1)	1	1.05 (0.97, 1.13)	0.211
hexanoylglycine	1	1.03 (0.98, 1.08)	0.228
N−formylmethionine	1	1.05 (0.97, 1.13)	0.249
homoarginine	1	1.02 (0.98, 1.06)	0.251
bilirubin (Z,Z) / etiocholanolone glucuronide	1	1.02 (0.98, 1.06)	0.297
succinimide	1	1.05 (0.95, 1.16)	0.301
N−acetyl−aspartyl−glutamate (NAAG)	1	0.99 (0.97, 1.01)	0.35
4−cholesten−3−one	1	0.95 (0.85, 1.06)	0.362
citrulline / dimethylarginine (SDMA + ADMA)	1	0.95 (0.85, 1.06)	0.369
4−oxo−retinoic acid	1	1.05 (0.94, 1.17)	0.409
uridine	1	1.04 (0.94, 1.16)	0.41
betaine	1	0.98 (0.92, 1.04)	0.418
phosphate / tyrosine	1	1.08 (0.89, 1.31)	0.424
serine	1	1.03 (0.96, 1.10)	0.429
4−guanidinobutanoate	1	0.99 (0.96, 1.02)	0.429
5,6−dihydrothymine	1	1.05 (0.93, 1.18)	0.442
creatine / carnitine	1	1.05 (0.93, 1.17)	0.442
erythronate*	1	0.97 (0.90, 1.05)	0.47
hypotaurine	1	1.05 (0.92, 1.19)	0.481
N−alpha−acetylornithine	1	1.03 (0.95, 1.11)	0.483
phosphate / uridine	1	0.96 (0.86, 1.08)	0.499
N−acetylhistidine	1	1.01 (0.97, 1.06)	0.503
creatine	1	1.04 (0.92, 1.17)	0.515
sphinganine−1−phosphate	1	1.04 (0.92, 1.17)	0.524
sphingosine 1−phosphate	1	1.04 (0.91, 1.19)	0.543
pregnenediol sulfate (C21H34O5S)*	1	1.04 (0.92, 1.18)	0.547
2'−deoxyuridine	1	0.98 (0.90, 1.06)	0.558
S−adenosylhomocysteine (SAH) / leucine	1	0.98 (0.91, 1.06)	0.598
beta−alanine	1	1.02 (0.95, 1.09)	0.6
O−sulfo−L−tyrosine	1	0.97 (0.88, 1.08)	0.617
5−methyluridine (ribothymidine)	1	1.01 (0.98, 1.04)	0.619
choline	1	1.03 (0.92, 1.15)	0.62
beta−hydroxyisovalerate	1	0.98 (0.88, 1.08)	0.637
3beta−hydroxy−5−cholestenoate	1	1.01 (0.97, 1.05)	0.645
thyroxine	1	0.97 (0.86, 1.11)	0.689
2'−deoxyuridine / cytidine	1	1.02 (0.93, 1.11)	0.705
N−palmitoylglycine	1	0.99 (0.95, 1.03)	0.714
uridine / pseudouridine	1	0.98 (0.86, 1.11)	0.716
3−methoxytyrosine	1	0.98 (0.86, 1.12)	0.763
beta−citrylglutamate	1	1.00 (0.97, 1.04)	0.77
N−acetylputrescine / (N(1) + N(8))−acetylspermidine	1	0.99 (0.91, 1.07)	0.772
cortisol	1	0.98 (0.87, 1.11)	0.773
glycine / serine	1	0.99 (0.90, 1.09)	0.792
cholesterol / cortisol	1	1.02 (0.90, 1.15)	0.803
sebacate (C10−DC)	1	1.01 (0.92, 1.11)	0.858
hydantoin−5−propionate	1	1.01 (0.90, 1.13)	0.886
N−acetylglucosaminylasparagine	1	1.00 (0.91, 1.09)	0.966
cysteine−glutathione disulfide	1	1.00 (0.95, 1.05)	0.982
orotate	1	1.00 (0.98, 1.03)	0.983
2−hydroxyglutarate	1	1.00 (0.88, 1.14)	0.995

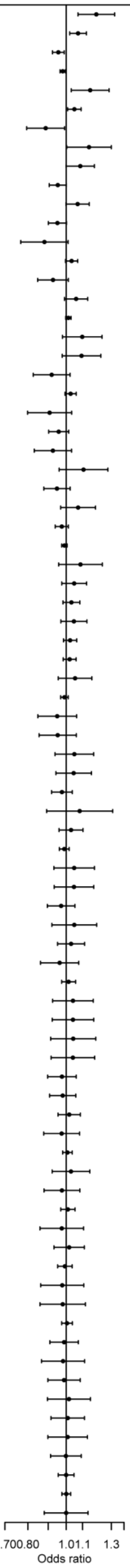

SUPPLEMENTARY FIGURE S3 Complete single-sentinel Mendelian randomization forest plot for Pain_Lowback, which was analyzed separately as an IVDD-related pain phenotype rather than as an anatomical IVDD outcome. Each row represents one metabolite–outcome test using a single sentinel SNV and the Wald-ratio estimator. Points show ORs per 1-SD increase in genetically proxied metabolite or metabolite-ratio level, and horizontal bars show 95% CIs. The vertical dashed line marks the null value (OR = 1). The N-SNV column (= 1 for all rows) indicates that each estimate is based on a single instrument. An asterisk indicates transformed exposure units: 1 SD of log-normalized values for metabolites and 1 SD of inverse rank-normalized values for metabolite ratios. CI, confidence interval; IVDD, intervertebral disc degeneration; MR, Mendelian randomization; N-SNV, number of single-nucleotide variants; OR, odds ratio; Pain_Lowback, Low back pain; SD, standard deviation; SNV, single-nucleotide variant.
